# A transcriptional switch controls meiosis

**DOI:** 10.7554/eLife.31911

**Published:** 2017-10-24

**Authors:** A Elizabeth Hildreth, Karen M Arndt

**Affiliations:** Department of Biological SciencesUniversity of PittsburghPittsburghUnited States

**Keywords:** meiosis, kinetochore, gene regulation, uORF translation, budding yeast, transcription, *S. cerevisiae*

## Abstract

A key protein involved in the segregation of meiotic chromosomes is produced 'just in time' by the regulated expression of two mRNA isoforms.

**Related research article** Chia M, Tresenrider A, Chen J, Spedale G, Jorgensen V, Ünal E, van Werven FJ. 2017. Transcription of a 5' extended mRNA isoform directs dynamic chromatin changes and interference of a downstream promoter. *eLife* **6**:e27420. doi: 10.7554/eLife.27420

**Related research article** Chen J, Tresenrider A, Chia M, McSwiggen DT, Spedale G, Jorgensen V, Liao H, van Werven FJ, Ünal E. 2017. Kinetochore inactivation by expression of a repressive mRNA. *eLife* **6**:e27417. doi: 10.7554/eLife.27417

To grow and develop properly, cells must control the timing of critical events. This means that the proteins that drive these events need to be active at specific times and, in many cases, this is achieved by precisely regulating when the genes that encode these proteins are transcribed. Now, in two papers in eLife, Elçin Ünal, Folkert van Werven and colleagues at the University of California at Berkeley and the Francis Crick Institute report a transcriptional switch that controls the timing of a critical event in the development of the yeast *Saccharomyces cerevisiae* (Chen et al., 2017; Chia et al., 2017).

Meiosis is the specialized form of cell division that produces new cells with half as many chromosomes as the parent cell. This process must be precise because too many or too few chromosomes in the new cells can lead to problems. In humans, where meiosis is used to make the egg and sperm cells, these problems include miscarriage or birth defects.

One key player that ensures chromosomes are segregated properly is the kinetochore. This protein complex acts as a molecular bridge between the chromosomes and the microtubule filaments that physically separate pairs of chromosomes during cell division (Duro and Marston, 2015). The kinetochore assembles, disassembles and reassembles during different stages of meiosis (Miller et al., 2012). During the first stage, which is called prophase, homologous chromosomes 'recombine' to exchange genetic information (Figure 1A): the kinetochore must disassemble during this stage to prevent the chromosomes being separated too soon. The expression of a protein called Ndc80, which is a key subunit within the kinetochore, drops when the meiotic kinetochore needs to dismantle, suggesting that the availability of Ndc80 acts as a molecular switch that controls whether kinetochores assemble or disassemble (Meyer et al., 2015; Chen et al., 2017).

**Figure 1.**
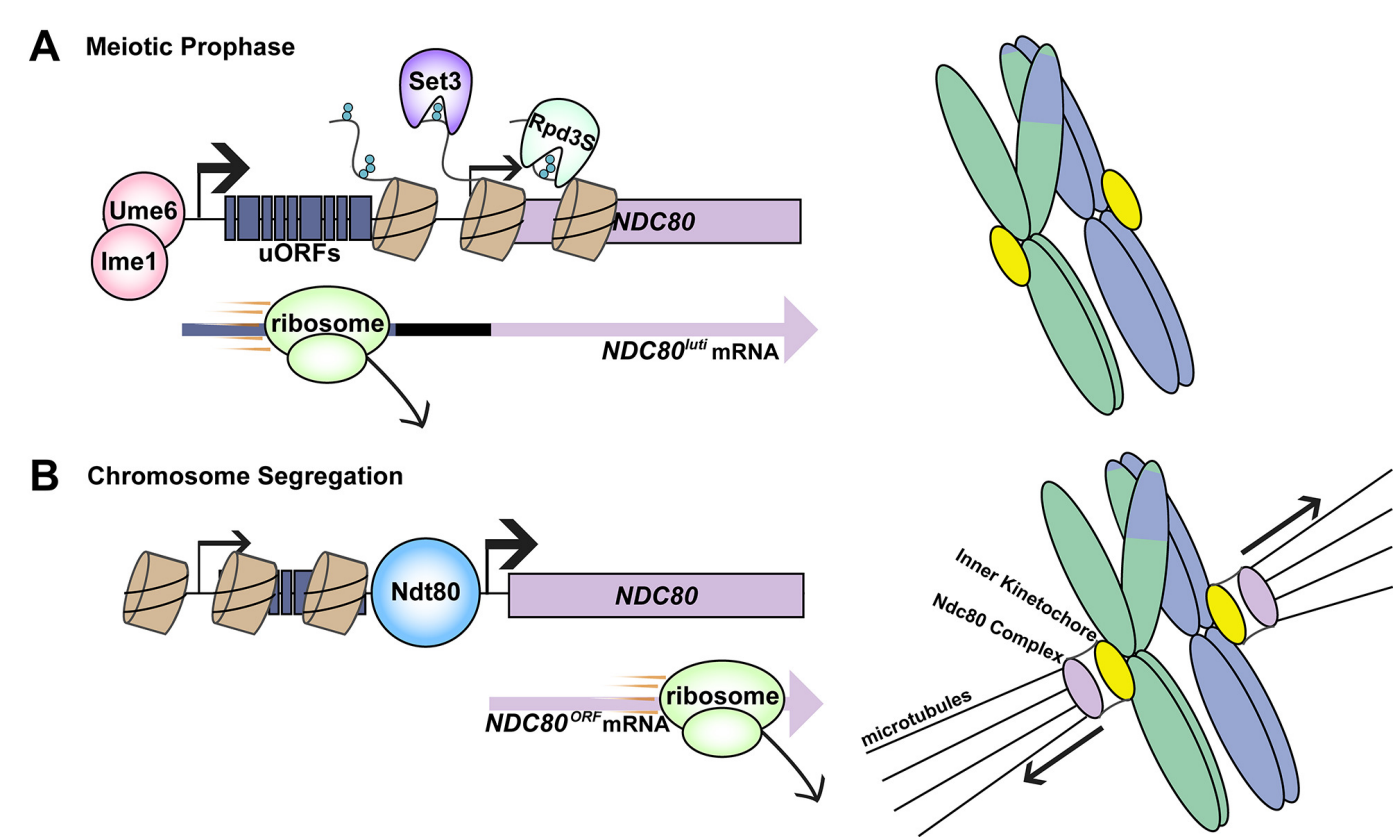
Transcriptional switching between two mRNA transcripts regulates the assembly of the kinetochore during meiosis. (**A**) During prophase, homologous chromosomes undergo recombination to exchange genetic information (right). The kinetochore must be dismantled during this time so that chromosomes do not prematurely separate. Cells do this by limiting the availability of a subunit of the kinetochore called Ndc80 (left top). First, Ume6-Ime1 binds to an upstream promoter (thick black arrow) and the DNA is transcribed to produce an extended mRNA transcript named *NDC80^luti^*. This process also results in the deposition of methylation marks (small blue circles) on histones (brown cyclinders). Enzymes called histone deacetylases (Set3 and Rpd3S) bind these marks and deacetylate the histones; the end result is to prevent the gene for Ndc80 (lilac rectangle) from being transcribed to produce a short mRNA transcript called *NDC80^ORF^*. Reduced transcription from the *NDC80^ORF^* promoter is indicated by the thinner black arrow. Additional regulation comes from upstream open reading frames (uORFs) in the extended transcript; the translation of these regions by the ribosome prevents translation reinitiation taking place at the *NDC80* start codon (left bottom). (**B**) Following prophase, the kinetochore reassembles for chromosome segregation (right). Another transcription factor, Ndt80, binds the canonical *NDC80* promoter (left top), and the DNA is transcribed to produce the shorter mRNA transcript. Translation of this transcript produces functional Ndc80 protein (left bottom), allowing the kinetochore to assemble and the chromosomes to segregate. Black arrows indicate direction of movement.

In the first paper – which has Jingxun Chen and Amy Tresenrider as joint first authors – the researchers confirmed why the production of Ndc80 must be lowered until just before the stage when the chromosomes segregate (Chen et al., 2017). They saw that, if the gene for Ndc80 in yeast was kept activated throughout meiosis, many cells showed abnormal chromosome segregation.

Earlier studies had revealed that the gene for Ndc80 gives rise to two distinct mRNA transcripts (Brar et al., 2012). Chen et al. now show that the longer of these two transcripts *–* an extended isoform called *NDC80^luti^* – is specific to meiosis, and predominates during prophase. They also show that the gene for Ndc80 has two promoters, one for each transcript, and that the promoter for the extended isoform is recognized by an early meiosis transcription factor complex called Ume6-Ime1 (Figure 1A).

In the second paper, which has Minghao Chia as the first author, the researchers report that switching on the transcription of the extended isoform leads to chemical modifications of chromatin across the downstream promoter. These modifications likely recruit enzymes known as histone deacetylases that modify chromatin in a way that prevents the transcription of the shorter mRNA, which is called *NDC80^ORF^* (Jensen et al., 2013; Chia et al., 2017).

Intriguingly, the extended transcript contains nine short upstream open reading frames (uORFs) that are, in fact, translated by the protein synthesis machinery (Brar et al., 2012). The translation of the uORFs in the extended isoform effectively silences production of the Ndc80 protein by preventing translation of the downstream *NDC80* coding region. This phenomenon occurs at other uORF-containing genes as well (Hinnebusch et al., 2016). The combined transcriptional and translational interference limits the production of Ndc80 protein in a way that is readily reversible.

Once recombination is complete, the kinetochore must reassemble so that the chromosomes can be segregated. Binding of a later meiotic transcription factor, Ndt80, switches on the expression of the shorter mRNA as the cell exits prophase. Importantly, the repressive chromatin structure established by transcription of the longer mRNA isoform does not block the Ndt80 binding site (Chia et al., 2017). Moreover, the shorter transcript does not contain the uORFs, and so it produces functional Ndc80 protein that can be assembled into the kinetochore (Figure 1B).

Ndc80 is also crucial when chromosomes segregate during mitosis, when cells divide to create daughter cells with the same number of chromosomes as the parent cell. During this kind of cell division in yeast, the expression of the short isoform is ensured by the Ume6 transcription factor repressing the expression of the long isoform. This is because, when Ime1 is absent, Ume6 functions to repress transcription. These observations raise the question of how cells eliminate the existing pools of Ndc80 as they enter prophase in meiosis. Future studies will now need to determine if and how protein degradation contributes to rapid depletion of Ndc80.

These two papers complement previous studies on the importance of proper gene expression in meiotic control. For example, the expression of *IME4,* an important gene for entry into meiosis, is controlled in yeast by a different type of transcriptional switch in which transcription of the coding strand of DNA is repressed by transcription of the opposite, noncoding strand until the gene’s protein is needed (Hongay et al., 2006). The data used to identify the two *NDC80* transcripts also reveal that approximately 190 meiotic genes have extended transcripts like *NDC80^luti^* (Brar et al., 2012; Chen et al., 2017; Chia et al., 2017). A goal for the future will be to characterize the expression of these genes, which might confirm that interfering with transcription and translation are general regulatory trends in the tuning of gene expression during meiosis.
